# Changes in blood glucose and metabolism in hyperuricemia mice

**DOI:** 10.1515/biol-2022-1057

**Published:** 2025-03-11

**Authors:** Fan Yang, Yan Zhao, Xingsan Li, Fengge Lou

**Affiliations:** Clinical Pathological Diagnosis Center, Qiqihar Medical University, Qiqihar, 161006, China; College of Public Health, Qiqihar Medical University, Qiqihar, 161006, China

**Keywords:** hyperuricemia, mice, blood glucose, metabolic patterns, streptozotocin

## Abstract

The aim of this study is to explore the changes in blood uric acid level, blood glucose, and metabolism in hyperuricemia (HUA) mice. The urate oxidase gene of C57BL/6J mouse is knocked out by targeted gene modification technology, and a spontaneous HUA mouse model is established. In the experiment, 20 urate oxidase gene knockout homozygous and wild type C57BL/6J mice are selected to construct the experimental group (EG) and the control group (CG), and the mice in both groups receive multiple intraperitoneal injections of low dose streptozotocin (STZ) solution. The changes in metabolic related indicators such as blood glucose, pancreatic β cell function, water intake, urination, food intake, and body weight are observed and compared between the EG and CG mice. Baseline indicators other than body weight between the two groups of mice before the experiment have no significant difference, *P* > 0.05. After the injection of STZ solution, body weight between the two groups has significant difference, *P* < 0.05. Before the experiment and less than 19 days after the start of the experiment, daily water intake and urine output between the two groups of mice have no significant difference, *P* > 0.05. After the experiment reaches 19 days, two groups’ water intake and urine output have significant difference, *P* < 0.05. Daily food intake between the two groups of mice has no significant difference, *P* > 0.05. Before the injection of STZ solution, fasting blood glucose levels between the two groups of mice has no significant difference, *P* > 0.05. The plasma insulin level of the EG mice was higher than that of the CG mice, with significant difference (*P* < 0.05). At the same time, the Homeostasis Model Assessment of Insulin Resistance index and fasting blood uric acid level of the EG mice were overall higher than those of the CG mice, with significant difference (*P* < 0.05). From the seventh day after the injection of STZ solution, the random blood glucose level, fasting blood glucose level, fasting insulin level, and blood uric acid level of the EG mice were higher than those of the CG mice, with significant difference (*P* < 0.05). For spontaneous HUA mice, the continuous increase in blood uric acid level caused by the disease may cause the increase in blood sugar content, thus promoting the occurrence of diabetes. Second, the content of uric acid in spontaneous HUA mice is maintained at a high level, which will bring or aggravate the damage of pancreatic islet β cells.

## Graphical Abstract

Study investigates the changes in blood uric acid, glucose, and metabolism in HUA mice using a knockout model. Mice with spontaneous HUA show increased blood sugar, insulin resistance, and β-cell damage after STZ injection. Higher uric acid levels may promote diabetes and exacerbate β-cell damage. Significant differences in metabolic indicators were observed between EG and CG.

**Figure:**
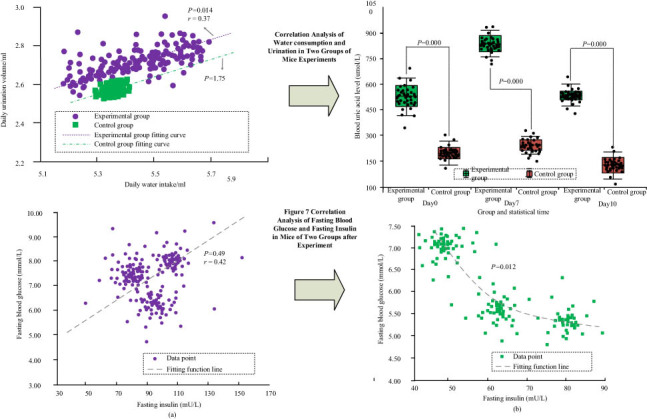

## 1 Introduction

Hyperuricemia (HUA) is caused by the disorder of purine metabolism, the decrease in uric acid secretion or the increase in excretion, which is manifested by the increase in blood uric acid level. According to the epidemiological survey, the incidence rate of HUA is on the rise, and more people are diagnosed with this disease [1,2]. The global prevalence of HUA varies by regional and lifestyle differences, with a general estimate of 5–20%. The global prevalence of diabetes is about 10% in adults and is expected to increase to 1.3 billion by 2045. HUA and diabetes often intersect with other metabolic diseases such as obesity, hypertension, and hyperlipidemia. HUA can induce many diseases, such as gout, kidney stone disease, kidney disease, and cardiovascular disease. HUA and diabetes share the same pathogenesis and pathophysiological process. The relationship between the two can be understood from the following aspects. First, insulin resistance is a common cause of both, which can lead to diabetes and HUA [3]. Second, HUA may be an independent risk factor of insulin resistance and closely related to the onset of diabetes [4]. Moreover, hyperglycemia may promote the excretion of uric acid salt, which may aggravate the symptoms of HUA [5,6,7]. In addition, HUA and diabetes are both pathophysiological processes characterized by oxidative stress and inflammatory reaction, which jointly promote the occurrence and progress of cardiovascular diseases [8,9,10]. Therefore, it is of great significance to study the relationship between HUA and changes in blood glucose and metabolism. In recent years, research have expressed that HUA is also closely related to metabolic diseases such as diabetes. The changes in blood glucose and metabolism may have an important impact on the course and treatment of HUA patients.

The research by Su et al. showed that 762 patients with type 2 diabetes were found during an average follow-up period of 2.9 years. Their cross-baseline HUA was proportional to the corresponding multivariate adjusted risk level of diabetes. In addition, the increase and decrease in uric acid level of participants in the first year were closely related to the increase and decrease in diabetes risk [11,12]. The research results proved that it is of great significance to pay attention to and reduce the risk of HUA patients developing diabetes during clinical diagnosis and treatment [13]. Gao’s research team designed a Chinese herbal formula and carried out an experiment to test the effect of this prescription on gout and HUA. The research outcomes denoted that the gout and HUA of the control group (CG) mice who did not take the drug were not significantly improved, while those of the experimental group (EG) mice who took the drug were alleviated to a certain extent [10]. The research on HUA, blood glucose, and metabolic changes is yet to be deepened. Some research results denoted that insulin resistance in HUA patients was related to gender, age, family history, habits, and other factors to a certain extent. In addition, some studies indicated that the HUA mouse model was abnormal in blood glucose metabolism, suggesting that there was a potential relationship between HUA and diabetes. However, these studies still have many shortcomings, such as small sample size, single research method or only focusing on one aspect of indicators, which cannot fully reflect the relationship between HUA and blood glucose and metabolic changes. Therefore, systematic research on HUA mice can not only deepen the understanding of pathophysiological processes but also help to explore new therapeutic strategies.

Given this, it is necessary to conduct more in-depth research using animal models. Mice, as experimental animals, have advantages such as economy, ease of operation, and similar physiological and biochemical indicators to humans, and have been widely used in biomedical research [14]. By constructing an HUA mouse model, it can directly observe and measure the effects of HUA on blood glucose and metabolic changes, and provide effective suggestions for clinical treatment. In this study, the targeted gene modification technique is used to construct the urate oxidase gene knockout model of C57BL/6J mice to obtain spontaneous HUA mice. To explore the relationship between HUA and changes in blood glucose and metabolism, the changes in blood glucose and metabolism of homozygous and wild-type mice under the action of streptozotocin (STZ) are compared.

The research on the relationship between HUA and blood glucose and metabolic changes has several limitations. First, many studies have small sample sizes, which may lead to inaccurate or unreliable results and limit the generalizability of the findings. Second, single research methods are often employed, failing to comprehensively and multi-dimensionally assess the complex relationship. For example, only one aspect of indicators is focused on, neglecting other potentially important factors. Third, some studies on HUA mouse models have limitations in reflecting the real situation, such as abnormal blood glucose metabolism in the models but not fully representing the actual relationship between HUA and diabetes. Moreover, current research lacks a more systematic and in-depth exploration, and the understanding of pathophysiological processes is still insufficient. Overall, these shortcomings call for more comprehensive, large-scale, and multi-method research to better understand the relationship between HUA and blood glucose and metabolic changes.

This study is mainly divided into three sections. Section 1 is used to describe the research background of the relationship between HUA and blood glucose metabolism, and analyze the purpose of this study. Section 2 is used to design a mouse model and experimental protocol. Section 3 is used to organize the results of mouse experiments and draw analytical conclusion.

## 2 General information and methods

### 2.1 Experimental equipment and material preparation

This experiment requires multiple biological and solution materials, and the preparation methods are as follows: 40 C57BL/6J mice are cultivated and randomly divided into an EG and CG, each containing 20 mice. Mice begin participating in the experiment when their growth cycle reaches 6 weeks of age. Take two clean beakers, weigh out a portion of 1.94 g citric acid material in a beaker, and add 100 mL of distilled water to the beaker. Stir well to obtain solution A. 2.97 g sodium citrate and 100 mL of distilled water are added to another beaker, and stirred thoroughly to obtain solution B. The solutions of the two beakers are mixed to obtain a citric acid buffer with a pH range of 4.2–4.5. 20 mg of STZ powder is weighted with an electronic scale, the STZ powder is put into a test tube, and citric acid buffer solution is added until 5 mL of solution is obtained. In this study, STZ is dissolved in citric acid buffer at a dose of 40 mg/kg and administered to mice via intraperitoneal injection once daily for 5 consecutive days. A standard sample is taken out using the enzyme-linked immunosorbent assay (ELISA) kit, centrifuged at a speed of 10,000 rpm for 30 s, diluted with 1 mL of sample, and repeatedly blown five times with a gun to obtain the standard solution. The concentrated washing solution is diluted with deionized water in a ratio of 1:25, then 240 mL of deionized water and 10 mL of concentrated washing solution are placed in a clean beaker, stirred well and kept aside. The biotin labeled antibody dilution solution is diluted in a ratio of 1:100 and stirred for later use. Horseradish peroxidase labeled avidin working solution is prepared at a ratio of 1:100. The serum samples were diluted in a ratio of 1:200. First, 52 µL of serum was added to a 45 µL dilution, then 15 µL was mixed and added to 285 µL of thinner.

The following equipment and materials are needed in the experiment: glucose meter, mouse fixator, desktop high-speed centrifuge, electronic weighing instrument, pure water meter, pH meter, micropipette, refrigerator at 4°C and −20°C, disposable sterile syringe and sterile insulin syringe, microplate meter, scissors and tweezers of various models, microscope, full-automatic blood biochemical analyzer, desktop ice maker, desktop low-temperature ultracentrifuge mice insulin enzyme-linked immunosorbent kit, blood glucose test strip, STZ, insulin antibody, distilled water, 0.9% sodium chloride solution, citric acid and sodium citrate, chloral hydrate solution, 4% formaldehyde solution, common feed for SPF mice, common bedding for SPF mice, C57BL/6J mice.


**Ethical approval:** The research related to animal use has been complied with all the relevant national regulations and institutional policies for the care and use of animals.

### 2.2 Mouse model construction and specimen collection

Wild type mice and urate oxidase gene knockout homozygous C57BL/6J mice are adaptively fed for 7 days. The former is divided into EG and the latter into CG. After 7 days, two groups of mice are injected intraperitoneally with a concentration of 40 mg/kg STZ solution, with an injection frequency of one time per day, for 5 consecutive days. All mice are fed from 9:00 to 10:00 am every day, and the feed is always distributed in a fixed amount regardless of whether the feed was consumed the previous day. At the same time, sufficient room temperature distilled water was provided for mice to drink throughout the entire process, and the bedding of all mice was replaced every 3 days. The lighting in the breeding environment is turned on at 8am every day and turned off at 8 pm every night to simulate the diurnal changes of mice in their natural growth environment.

During the experiment, venous blood needs to be collected at the same time before STZ injection, and on the 7th, 10th, and 28th days after injection. Mice need to undergo 8–12 h of fasting before collecting venous blood. 0.3 mL of blood is collected from the tail vein of mice and let it stand at room temperature for 1–2 h to allow the serum to precipitate. The serum is centrifuged at 3,500 rpm for 15 min and approximately 150 µL of serum are collected for future use. After 28 days of STZ solution injection, chloral hydrate solution is injected into the abdominal cavity of mice for anesthesia, and the abdominal cavity is opened quickly with tweezers and tissue scissors, to remove the pancreas. The pancreas is gently peeled off and ophthalmic scissors are used to remove surface adipose tissue. The pancreas is fixed in a 4% neutral formaldehyde solution for 24 h, then made to undergo alcohol gradient dehydration treatment, and finally immersed in wax to make a wax block, which is stored for future use.

### 2.3 Testing indicators and methods

**Figure 1:**
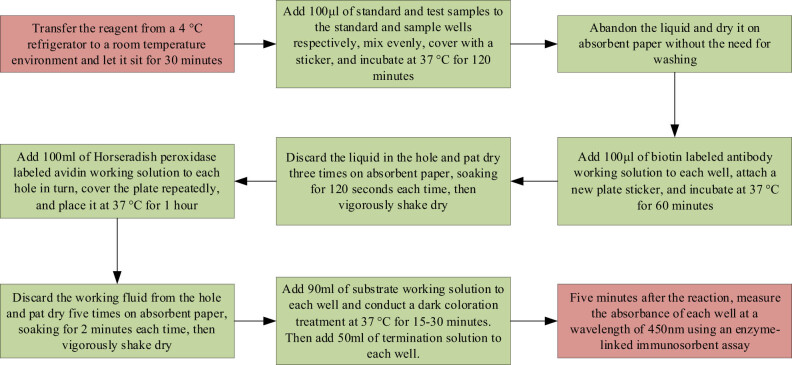
Flow chart for measuring fasting serum insulin content in mice.

The indicators that need to be tested in this study include mouse blood glucose levels and random tail vein blood glucose levels, fasting blood uric acid levels, fasting serum insulin content, body weight, daily water intake, daily urination, and daily food intake. The mouse fasting blood glucose levels and random tail vein blood glucose of mice are measured by Roche Glucose meter, and the fasting blood uric acid value of mice is measured by automatic biochemical analyzer. The fasting serum insulin content of mice is measured using ELISA, as shown in Figure 1.

### 2.4 Immunohistochemical staining method for pancreatic specimens

**Figure 2:**
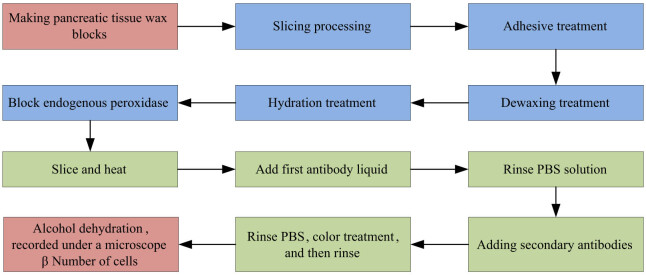
Immunohistochemical staining of mouse pancreatic specimens.

The process of immunohistochemistry staining for mouse pancreatic specimens is shown in Figure 2. The first step is to make a wax block, which includes sampling, dehydration, transparency, wax immersion, and embedding. Materials: The pancreas tissue of mice is placed in 4% neutral formaldehyde solution for 24 h, and then taken out and cut it into 2 mm tissue blocks with a scalpel. Dehydration: Pancreatic tissue blocks are soaked in a concentration of 30, 50, 70, 80, 90% anhydrous ethanol solution for dehydration. Transparent: The dehydrated tissue is immersed into xylene solution twice for 30 min. Soaking in wax: The tissue is soaked in soft waxes I, II, and III for 60, 120, and 120 min, respectively. Embedding: The immersed tissue is placed in the middle of the embedding frame and the embedding frame is placed on a cooling table for backup. Next is the slicing process. First, a rotary slicer is used to slice the prepared pancreatic tissue into paraffin sections with a thickness of 5 microns. Then, the slices are placed on a glass slide with adhesive, to flatten them. Excess water is absorbed by filter paper, and then dried in an oven at a constant temperature of 40°C. Next the slides containing pancreatic tissue are sequentially placed in xylene I for 5 min and xylene II for 5 min for dewaxing. Subsequently, it is soaked in anhydrous ethanol, 95, 90, 80, and 70% ethanol for 5, 3, 2, and 1 min, respectively, and then washed with phosphate buffered salt (PBS) solution for 1–2 min. The next step is to incubate the slices with 3% hydrogen peroxide deionized water for 15 min, and then rinse with PBS solution. Then, the slices are placed in a PBS solution, heated at high temperature and low temperature in a microwave oven for 1 and 19 min, respectively and cooled at room temperature and rinsed with PBS solution. Afterwards, the first antibody is incubated in a 37°C incubator for 60 min, rinsed with PBS solution, and the excess water was absorbed. Then, the enzyme secondary antibody polymer is added. It is incubated in a 37°C incubator for 30 min, and rinsed with PBS solution. Next the staining solution is added and the staining of the cells is observed. After staining, the staining is terminated and the cells are washed with distilled water. Finally, the staining results of mouse pancreatic islets are analyzed under an optical microscope and the percentage of brown area (i.e., beta [β] cells) in mouse pancreatic islets is calculated. The percentage of cells is used to represent the number of β cells in a single pancreatic islet.

In addition, quantitative analysis of the morphology and function of pancreatic islets is required. It mainly includes three aspects: measurement of pancreatic islet area, counting of pancreatic β cells, and evaluation of pancreatic β cell function. Islet area measurement: Image analysis software (such as ImageJ) is used to measure the total area of the pancreas in each slice and its ratio to the total area of the pancreas is calculated to evaluate the relative size of the pancreas. Pancreatic β cell count: Under an optical microscope, the pancreatic β cells in each slice are counted and the number of β cells per square millimeter of pancreatic islets is calculated to evaluate the density of pancreatic β cells. Assessment of pancreatic β cell function: The functional status of pancreatic β cells is evaluated by measuring the number and distribution of insulin secreting granules, as well as the expression levels of insulin synthesis and secretion related proteins (such as proinsulin, glucokinase, etc.) within β cells.

To investigate the interactions between uric acid levels, insulin sensitivity, and glucose metabolism, as well as the key roles of specific gene variations in these processes, the study set up experimental models of different strains of mice (C57BL/6J, BALB/c, DBA/2) to observe and compare their differences in uric acid levels, insulin sensitivity, and glucose metabolism, while detecting the expression levels of related genes. C57BL/6J is used as an HUA model, BALB/c is used as a normal CG, and DBA/2 shows a positive effect of low uric acid levels. Through comparative analysis, it is hoped to reveal the important role of uric acid insulin axis in diabetes and its complications.

### 2.5 Statistical methods

All data in the study are processed using Python 3.0 language, and the measurement data are presented in the form of mean value ± standard deviation, and *T* difference significance testing is required. The countable data in the study are presented in the form of number of cases or proportion of cases, and \[{\chi }^{2}]\] significance test is required. The significance level of all difference significance tests is taken as 0.05, i.e., when the *P* value obtained from the difference significance test is less than 0.05, it is considered that the difference between the two groups of data is statistically significant. The significance level of the correlation analysis in the study is also set to 0.05.

## 3 Results

### 3.1 Baseline data of mice before the experiment and comparison of mouse weight before and after the experiment

The baseline data statistics of each group of mice before the experiment are displayed in Table 1. From Table 1, before the experiment, the *P* value of *T* or \[{\chi }^{2}]\] difference significant test for the data of each group of mice in terms of gender distribution, body length, daily drinking water, daily food intake, daily urination, and fasting glucose blood level was greater than 0.05, and it was considered that the data difference had no statistical significance. However, the *P* value of the *T*-test in terms of body weight between the two groups of mice was less than 0.05, indicating the difference had statistical significance.

**Table 1:** Baseline data of each group of mice before the experiment

Statistical items		EG	CG	*T*/ \[{\chi }^{2}]\]	*P*
Gender	Female	11	10	0.053	0.819
	Male	9	10		
Weight (g)	/	20.1 ± 1.9	23.4 ± 2.4	4.821	0.000
Body length (mm)	/	96.2 ± 7.4	97.4 ± 8.8	0.157	1.963
Growth time (d)	/	40.8 ± 2.5	41.2 ± 2.8	0.338	1.217
Daily drinking water volume (mL)	/	5.21 ± 0.47	5.40 ± 0.38	1.480	0.147
Daily food intake (g)	/	7.0 ± 1.6	6.8 ± 1.8	0.744	0.519
Daily urination volume (mL)	/	2.62 ± 0.29	2.59 ± 0.35	0.295	0.770
Glucose test – Fasting blood sugar level	/	7.48 ± 0.71	7.32 ± 0.69	0.723	0.474

The changes in body weight of the two groups of mice before and after the experiment are shown in Table 2. “Day0” in Table 2 represents the time before the experiment. “#” means *P* < 0.05 in the data of the same group of mice compared to the earliest data appearing in the chart. From Table 2, it can be observed that at each time before and after the experiment, the *T*-test *P*-values of the body weight data of the two groups of mice were all less than 0.05, indicating a statistically significant difference, and the overall weight of the CG mice was larger. Within the same group, the body weights of mice at any time after the start of the experiment were compared with the pre-experimental data, indicating *P* < 0.05.

**Table 2:** Comparison of body weight between two groups of mice before and after the experiment

Statistical items	EG (g)	CG (g)	*T*	*P*
Day0	20.1 ± 1.9	23.4 ± 2.4	4.821	0.000
Day7	20.5 ± 2.4	23.6 ± 2.1	4.452	0.000
Day10	20.3 ± 1.8	22.5 ± 1.9	2.315	0.035
Day13	19.5 ± 2.0	23.4 ± 2.1	5.148	0.000
Day16	19.9 ± 2.1	24.2 ± 2.3	5.756	0.000
Day19	20.3 ± 2.3	24.5 ± 2.6	5.420	0.000
Day22	20.1 ± 2.0	23.7 ± 2.5	4.963	0.000
Day25	20.0 ± 2.3	23.4 ± 2.1	4.890	0.000
Day28	20.2 ± 2.4	23.8 ± 2.4	5.257	0.000
Day31	19.8 ± 1.9	24.2 ± 2.2	5.359	0.000

At the end of the experiment, the effect of purinol on blood glucose and antioxidant levels in HUA mice was investigated. The study set up five EGs, CG A: healthy C57BL/6J mice that were not induced to HUA or treated with allopurinol. HUA group B: Knocking out the uric acid oxidase gene through targeted gene modification technology to establish an HUA mouse model, without receiving allopurinol treatment. Allopurinol treatment group (low-dose) C: HUA mice received low-dose allopurinol treatment (specific dose determined according to experimental design). Allopurinol treatment group (medium-dose) D: HUA mice received medium-dose allopurinol treatment (higher dose than low-dose group). Allopurinol treatment group (high-dose) E: HUA mice received high-dose allopurinol treatment (higher than the medium-dose group). All EG mice were kept under the same feeding conditions and dietary management during the experiment to ensure the reliability of the experimental results. The blood glucose levels, plasma lipid peroxide levels, and antioxidant levels were measured at the end of the experiment using standard biochemical analysis methods. The experimental results are shown in Table 3.

**Table 3:** Effects of allopurinol on blood glucose and antioxidant levels in HUA mice

EG	Plasma lipid peroxides (μmol/L)	SOD (U/mL)	Catalase (U/mL)	Glutathione (μg/mL)	Blood glucose level (mmol/L)
CG	1.50 ± 0.20	120.0 ± 10.5	15.5 ± 1.2	4.2 ± 0.3	10.5 ± 1.2
HUA group	2.20 ± 0.30	90.0 ± 8.5	12.0 ± 1.0	3.0 ± 0.2	14.0 ± 1.5
Allopurinol treatment group (low-dose)	1.80 ± 0.25	105.0 ± 9.5	13.5 ± 1.1	3.5 ± 0.3	12.0 ± 1.3
Allopurinol treatment group (medium-dose)	1.60 ± 0.20	115.0 ± 10.0	14.5 ± 1.2	3.8 ± 0.3	11.0 ± 1.1
Allopurinol treatment group (high-dose)	1.40 ± 0.15	125.0 ± 11.0	15.0 ± 1.3	4.0 ± 0.3	10.0 ± 1.0

**Figure 3:**
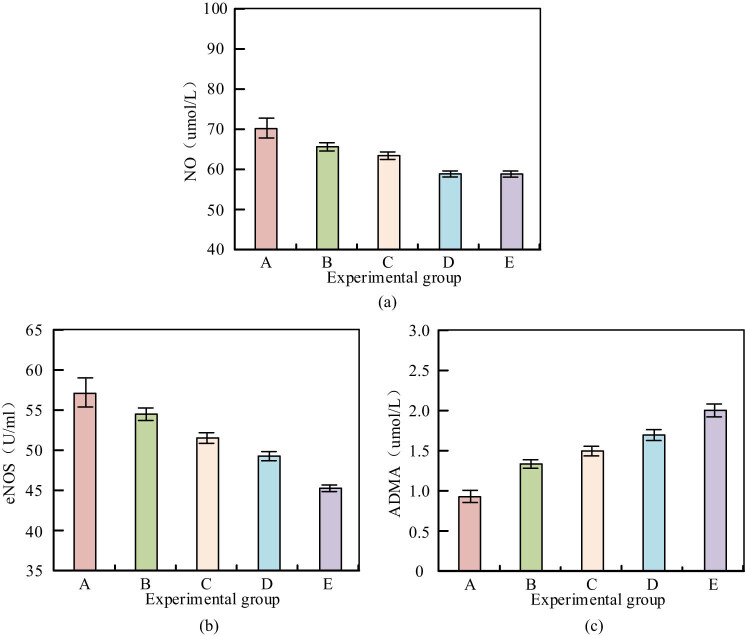
Expression of ADMA, eNOS, and NO in different EGs. (a) The expression of NO in different EGs. (b) eNOS expression in different EGs. (c) ADMA expression in different EGs.

According to the data in Table 3, allopurinol has a significant effect on blood glucose and antioxidant levels in mice with HUA. Compared with the CG, the plasma lipid peroxide levels in mice with HUA were significantly increased (2.20 ± 0.30 µmol/L vs 1.50 ± 0.20 µmol/L), while the levels of superoxide disruptase (SOD), catalase, and glutathione were significantly decreased, and blood glucose levels were also significantly increased (14.0 ± 1.5 mmol/L vs 10.5 ± 1.2 mmol/L). However, after treatment with allopurinol, these indicators showed significant improvement. As the dose of allopurinol increased, the levels of plasma lipid peroxides gradually decreased, while the levels of SOD, catalase, and glutathione gradually increased. Especially in the high-dose allopurinol treatment group, the plasma lipid peroxide levels were close to the CG (1.40 ± 0.15 µmol/L), and the blood glucose levels were significantly reduced to near normal levels (10.0 ± 1.0 mmol/L). These results indicated that allopurinol not only reduced uric acid but also improved the antioxidant capacity and blood glucose levels of HUA mice. These animals with high uric acid were likely to have atherosclerosis, so the expression of ADMA, eNOS, NO, and other related genes were also explored in five EGs. The results are shown in Figure 3.

From Figure 3, the NO level of CG A was the highest, about 70 nmol/L, eNOS expression exceeds 55 U/mL, and ADMA level was the lowest, about 0.8 µmol/L. The NO level in HUA group B significantly decreased to about 65 nmol/L, eNOS expression decreased to about 50 U/mL, and ADMA level significantly increased to about 1.3 µmol/L. These changes indicated that animals with HUA may have signs of endothelial dysfunction. After receiving allopurinol treatment, with the increase in dosage, the levels of NO and eNOS gradually increased, while the level of ADMA gradually decreased. The NO level of low-dose treatment group C was about 60 nmol/L, eNOS expression was about 48 U/mL, and ADMA level was about 1.2 µmol/L. The improvement was more significant in the medium-dose group D and the high-dose group E, especially in the high-dose group E, whose ADMA and NO levels were comparable to the CG, at 0.9 µmol/L and nearly 70 nmol/L, respectively. eNOS expression also increased to 52 U/mL. In summary, animals with HUA do show signs of endothelial dysfunction, characterized by significantly reduced eNOS expression and NO levels, and significantly increased ADMA levels. Allopurinol treatment could significantly improve these changes, and the higher the dose, the more obvious the effect.

### 3.2 Comparison of water consumption, food intake, and urination in each group of mice

**Figure 4:**
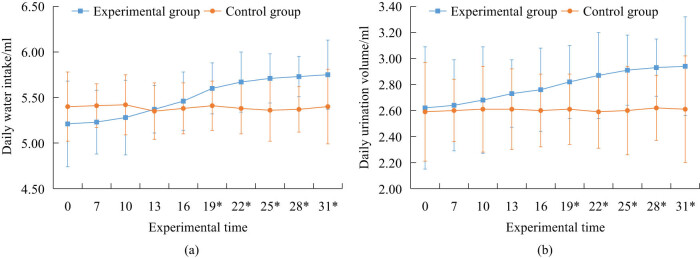
Water consumption and urine output of two groups of mice before and after the experiment. (a) Daily water intake. (b) Daily urination volume.

The water intake and urine output of two groups of mice were compared, as shown in Figure 4. The “*” represents a statistically significant difference in data between the two groups of mice at the same time. Observing Figure 3, the difference in daily water intake and daily urination between the two groups of mice was not statistically significant before and less than 19 days after the experiment began. However, after the experiment reached 19 days, daily water intake and daily urination between the two groups of mice had *P* < 0.05, and the values of the EG mice in both indicators were overall higher.

The comparison of food intake between the two groups of mice is shown in Table 3. Observing Table 4, the *P*-values of the daily food intake *T*-test for both groups of mice before and after the experiment were greater than 0.05 at each time, indicating that the difference was not statistically significant.

**Table 4:** Comparison of daily food intake between two groups of mice before and after the experiment

Statistical items	EG (g)	CG (g)	*T*	*P*
Day0	7.0 ± 1.6	6.8 ± 1.8	0.744	0.519
Day7	7.0 ± 1.8	6.9 ± 1.4	0.196	0.846
Day10	6.9 ± 1.4	6.9 ± 1.8	0.082	1.641
Day13	7.0 ± 1.5	6.8 ± 1.6	0.775	0.502
Day16	7.1 ± 1.6	7.0 ± 1.3	0.216	0.830
Day19	6.8 ± 1.5	7.1 ± 1.5	0.829	0.437
Day22	7.0 ± 1.8	7.1 ± 1.5	0.224	0.811
Day25	7.1 ± 1.7	7.1 ± 1.7	0.085	1.640
Day28	6.9 ± 1.5	7.0 ± 1.5	0.192	0.853
Day31	6.9 ± 1.8	6.9 ± 1.4	0.094	1.469

The study conducted 20 replicates and found that daily food intake fluctuated between 6.8 g and 7.1 g throughout the experimental period, and the difference between the groups was not significant at each time point (*P* > 0.05). There was therefore no significant difference in daily food intake between the two groups during the experimental period, which may indicate that the experimental conditions did not significantly influence on food intake in both groups or that the groups showed similar adaptations to the experimental conditions. The morphological and functional changes of islets before and after experiments in both groups of mice are shown in Table 5.

**Table 5:** Morphological and functional changes of islets before and after the experiments in the two groups of mice

Statistical items	Proportion of pancreatic islet area to total pancreatic area (%)		Number of β cells per square millimeter of pancreatic islets (cells/mm²)		Relative level of proinsulin expression	
	EG	CG	EG	CG	EG	CG
Day 0	1.5 ± 0.3	1.8 ± 0.4	250 ± 30	300 ± 40	0.8 ± 0.1	1.0 ± 0.2
Day 7	1.6 ± 0.2	1.7 ± 0.3	260 ± 25	310 ± 35	0.9 ± 0.1	1.1 ± 0.1
Day 10	1.5 ± 0.4	1.6 ± 0.2	255 ± 30	305 ± 30	0.85 ± 0.1	1.05 ± 0.1
Day 13	1.4 ± 0.3	1.7 ± 0.4	240 ± 20	300 ± 30	0.8 ± 0.1	1.0 ± 0.2
Day 16	1.5 ± 0.2	1.8 ± 0.3	250 ± 25	310 ± 35	0.9 ± 0.1	1.1 ± 0.1
Day 19	1.6 ± 0.3	1.9 ± 0.2	260 ± 30	320 ± 40	0.95 ± 0.1	1.15 ± 0.1
Day 22	1.5 ± 0.2	1.7 ± 0.3	250 ± 25	305 ± 30	0.9 ± 0.1	1.05 ± 0.1
Day 25	1.4 ± 0.4	1.6 ± 0.2	240 ± 20	300 ± 25	0.8 ± 0.1	1.0 ± 0.1
Day 28	1.5 ± 0.3	1.8 ± 0.4	250 ± 30	310 ± 35	0.9 ± 0.1	1.1 ± 0.1
Day 31	1.6 ± 0.2	1.9 ± 0.3	260 ± 25	320 ± 30	0.95 ± 0.1	1.15 ± 0.1

According to the data in Table 5, changes in the morphology and function of the pancreas in both groups of mice during the experiment can be observed. In terms of the proportion of pancreatic islet area to total pancreatic area, the EG was 1.5% on day 1, while the CG was 1.8%, indicating that the pancreatic islets in the CG were relatively larger. On the last day of the experiment (31st day), the proportion of EG increased to 1.6%, while the CG increased to 1.9%, indicating a more significant increase in pancreatic islet area in the CG. In terms of the number of pancreatic β cells, the EG had 250 cells/mm^2^ on the first day, while the CG had 300 cells/mm², indicating a higher density of β cells in the CG. On the 31st day, the number of β cells in the EG increased to 260 cells/mm², while the CG increased to 320 cells/mm², further confirming the increase in β cell density in the CG. On the expression level of proinsulin, the EG was 0.8 on the first day, while the CG was 1.0, indicating that the expression level of proinsulin in the CG was higher. On the 31st day, the expression level of EG increased to 0.95, while that of CG increased to 1.15, indicating a more significant increase in the expression level of proinsulin in the CG. In summary, the pancreatic islet area, number of β cells, and expression level of proinsulin in the CG were higher than those in the EG throughout the entire experimental period, and the growth trend was more pronounced. This may indicate that the pancreatic islet function in the CG is more active. Elevated uric acid levels have a significant impact on pancreatic β cell function and insulin secretion in mice with HUA. Research outcomes showed that compared to the CG, mice with HUA had a reduced number of pancreatic β cells and impaired β cell function, manifested by lower levels of proinsulin expression. This suggests that high uric acid may lead to dysfunction of β cells through some mechanism. Although the direct causal relationship needs further study and confirmation, there is a close relationship between high uric acid level and β cell dysfunction, suggesting that uric acid may be an important factor affecting the occurrence and development of diabetes.

### 3.3 Randomized blood glucose comparison of each group of mice

The random blood glucose statistical results at each time after the experiment are shown in Table 6. On observing Table 6, it can be found that after the injection intervention of the EG mice, the *P* values of the random blood glucose *T*-test of the two groups of mice at each testing time were far less than 0.05, indicating that there was significant difference. Regardless of whether it was the EG or the CG, within the same group, after the first injection intervention, the random blood glucose data measured each time had significant difference compared to the data from Day7.

**Table 6:** Comparison of random blood glucose levels between two groups of mice before and after the experiment

Statistical items	EG (mmol/L)	CG (mmol/L)	*T*	*P*
Day7	12.2 ± 2.5	9.2 ± 1.8	4.355	0.000
Day10	20.4 ± 4.2	10.7 ± 2.5	14.082	0.000
Day13	23.9 ± 4.5	11.4 ± 2.4	18.14.6	0.000
Day16	28.2 ± 4.1	12.5 ± 2.6	13.767	0.000
Day19	27.8 ± 3.8	13.6 ± 2.4	14.152	0.000
Day22	23.5 ± 4.3	13.7 ± 2.4	15.248	0.000
Day25	21.0 ± 4.0	12.4 ± 2.0	13.689	0.000
Day28	16.7 ± 3.1	10.1 ± 2.1	5.787	0.000
Day31	14.1 ± 2.4	7.9 ± 1.5	8.051	0.000

### 3.4 Comparison of fasting blood glucose and insulin in mice of each group

The statistical results of fasting blood glucose of the two groups of mice at several times after the experiment can be seen in Table 7. It was noted that the “mean relative increase” in Table 5 represents the increase in the mean fasting blood glucose of the EG mice relative to that of the CG mice at the same time. From Table 7, the difference between the fasting blood glucose data before the experiment had no significant difference (*P* > 0.05), but the fasting blood glucose data on Day 7 and Day 10 had significant difference (*P* < 0.05), and the value of the EG was larger than that of the CG as a whole.

**Table 7:** Comparison of fasting blood glucose levels of mice in the two groups before and after the experiment (Unit: mmol/L)

Group	Number of mice/group	Day0	Day7	Day10
EG	20	7.48 ± 0.71	7.03 ± 0.59	8.25 ± 0.84
CG	20	7.32 ± 0.69	5.42 ± 0.47	5.36 ± 0.41
*T*	—	0.723	9.545	13.827
*P*	—	0.474	0.000	0.000
Relative increase in mean value	—	2.14%	29.70%	35.03%

**Figure 5:**
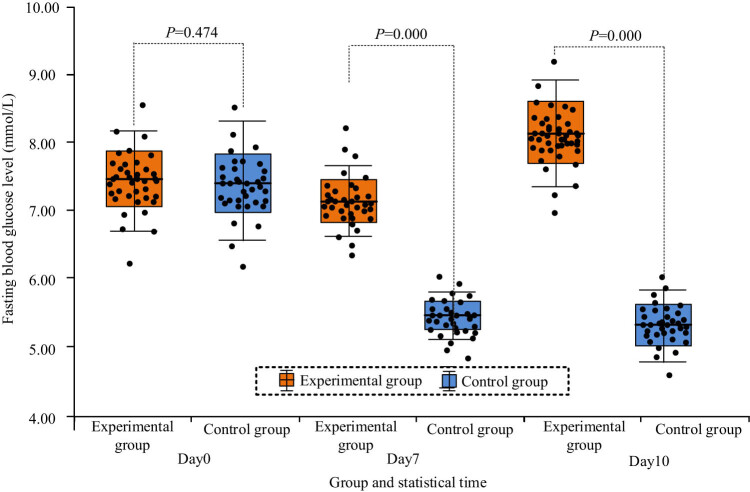
Distribution of fasting blood glucose at multiple times in two groups of mice.

The distribution of fasting blood glucose of mice in the two groups at several times after the experiment is shown in Figure 5. Observation of Figure 5 showed that the fasting blood glucose data of mice in the EG and CG all conformed to the normal distribution law. Before the experimental intervention, the distribution of fasting blood glucose in the two groups of mice was relatively dispersed, and on the tenth day after the start of the experiment, the distribution of fasting blood glucose data in the EG of mice became more dispersed, but after the start of the experiment, the distribution of this indicator in the CG of mice was more concentrated.

**Figure 6:**
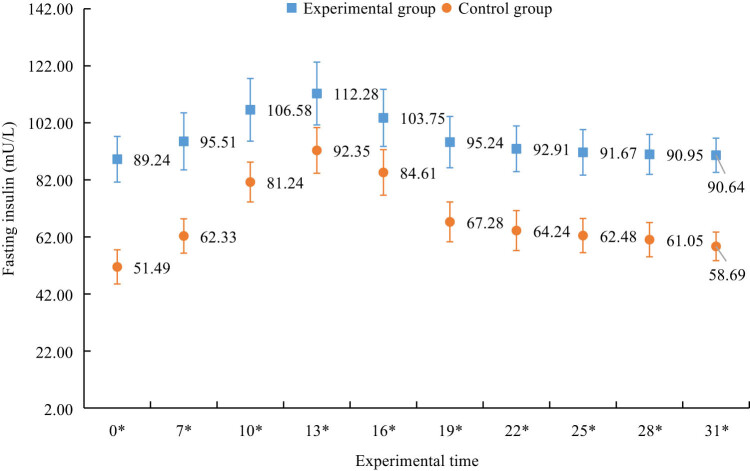
Fasting insulin levels of two groups of mice at multiple times after the experiment.

The statistical results of fasting insulin levels in two groups of mice at multiple times after the experiment are shown in Figure 6. Figure 6 shows that fasting insulin values between the two groups of mice before and after the experiment had significant difference (*P* < 0.05). The overall values of the EG were greater than those of the CG.

In Table 8, ↑ represents an increase in gene expression level or activity enhancement; ↓ indicates a decrease in gene expression level or weakened activity; ≈ indicates that the gene expression level or activity is similar to that of the normal CG. As an HUA model, C57BL/6J mice demonstrated the negative effects of elevated uric acid levels on insulin sensitivity and glucose metabolism, as well as changes in the expression of various related genes. In contrast, BALB/c mice in the CG had normal levels of uric acid, insulin sensitivity, and glucose metabolism, and the expression levels of related genes were relatively stable. DBA/2 mice demonstrated positive effects of low uric acid levels on insulin sensitivity and glucose metabolism, as well as corresponding gene expression changes. DBA/2 mice had the highest Homeostasis Model Assessment of Insulin Resistance (HOMA-IR index of 1.0 ± 0.2) and the most normal glucose metabolism (FPG of 4.5 ± 0.5 mmol/L), which was closely related to the normal or favorable variation of their gene expression levels. These data indicated that there were complex interactions between uric acid levels, insulin sensitivity, and glucose metabolism, and specific gene variations played a key role in them. Therefore, it is expected to develop a new treatment strategy for the uric acid insulin axis to prevent or treat diabetes and its complications by regulating the expression of related genes or uric acid level.

**Table 8:** Exact molecular mechanisms of insulin sensitivity and glucose metabolism in different strains of mice

Mouse strain	Uric acid level (μmol/L)	Insulin sensitivity (HOMA-IR index)	Fasting plasma glucose (FPG, mmol/L)	Specific gene variations and expression levels	*P* value
C57BL/6J	450 ± 50	3.5 ± 0.5	8.0 ± 1.0	SLC2A9 (GLUT9) ↑, IRS-1↓, PIK3R1↓, GLUT4↓, TNF-α↑, IL-6↑, PPARγ↓, ABCG2↓	<0.05
BALB/c	300 ± 30	2.0 ± 0.3	5.5 ± 0.5	SLC2A9 (GLUT9) ≈, IRS-1≈, PIK3R1≈, GLUT4≈, TNF-α≈, IL-6≈, PPARγ≈, ABCG2≈	<0.05
DBA/2	150 ± 20	1.0 ± 0.2	4.5 ± 0.5	SLC2A9 (GLUT9) ↓, IRS-1↑, PIK3R1↑, GLUT4↑, TNF-α↓, IL-6↓, PPARγ↑, ABCG2↑	<0.05

The data in Table 9 showed that the degree of β cell damage in the HUA group (EG) was higher than that in the CG, indicating that HUA may directly lead to β cell damage. For example, the C57BL/6J EG had a β cell injury score of 3, while the CG had a score of 1, which may indicate that high uric acid levels directly affect β cell function. Insulin resistance and HUA: The insulin resistance index (HOMA-IR) of the HUA group was generally higher than that of the CG, such as the HOMA-IR of 3.5 in the C57BL/6J EG group and 1.2 in the CG, indicating that HUA may be related to the development of insulin resistance. The blood glucose levels in the HUA group were generally higher, such as 10 mmol/L in the C57BL/6J EG and 5.2 mmol/L in the CG, which may reflect the adverse effects of HUA on blood glucose control. HUA was associated with changes in the levels of various biomarkers, such as SLC2A9 (GLUT9), IRS-1, PIK3R1, GLUT4, TNF-α, IL-6, PPARγ, and ABCG. These biomarkers help predict β cell dysfunction in individuals with HUA. The diversity index of gut microbiota in the HUA group was generally lower than that in the CG, such as 2.5 in the C57BL/6J EG group and 3.3 in the CG, indicating that HUA may affect the balance of gut microbiota, thereby affecting glucose metabolism and insulin sensitivity. In summary, HUA may directly damage pancreatic β cell function, increase insulin resistance, raise blood glucose levels, and affect metabolism by affecting the balance of gut microbiota. These findings suggest that early intervention against HUA may help prevent the development of diabetes, and regulating intestinal microbiota may become a new strategy to prevent or treat HUA and diabetes.

**Table 9:** Association between HUA and pancreatic β-cell function, insulin resistance, glycemia, and specific biomarkers, as well as the gut microbiota

Strain	Group	β-cell damage degree (score)	Insulin resistance index (HOMA-IR)	Blood glucose level (mmol/L)	Specific biomarker level (ng/mL)	Intestinal microbiota diversity index
C57BL/6J	EG	3	3.5	10	15	2.5
C57BL/6J	CG	1	1.2	5.2	5.1	3.3
BALB/c	EG	2.5	3.2	9.6	13	2.2
BALB/c	CG	0.8	1.0	4.5	3.5	3.2
DBA/2	EG	3.2	3.8	11.2	16	2.0
DBA/2	CG	1.1	1.1	4.8	4.3	3.6

### 3.5 Comparison of blood uric acid levels in each group of mice

The blood uric acid data of two groups of mice at multiple times before and after the experiment were compared. The statistical results are shown in Table 10. From Table 10, the *P* value of *T-*test of fasting blood glucose data before the experiment, on Day 7, and Day 10 was far less than 0.05, which indicated that it had significant difference (*P* < 0.05), and the value of the EG was larger than that of the CG as a whole.

**Table 10:** Comparison of blood uric acid levels between two groups of mice before and after the experiment (unit: µmol/L)

Group	Number of mice/group	Day0	Day7	Day10
EG	20	528.45 ± 69.33	849.75 ± 61.48	568.47 ± 19.44
CG	20	191.24 ± 32.57	251.39 ± 22.86	148.53 ± 26.71
*T*	—	19.688	41.241	58.328
*P*	—	0.000	0.000	0.000
Relative increase in mean value	—	176.44%	238.25%	283.78%

**Figure 7:**
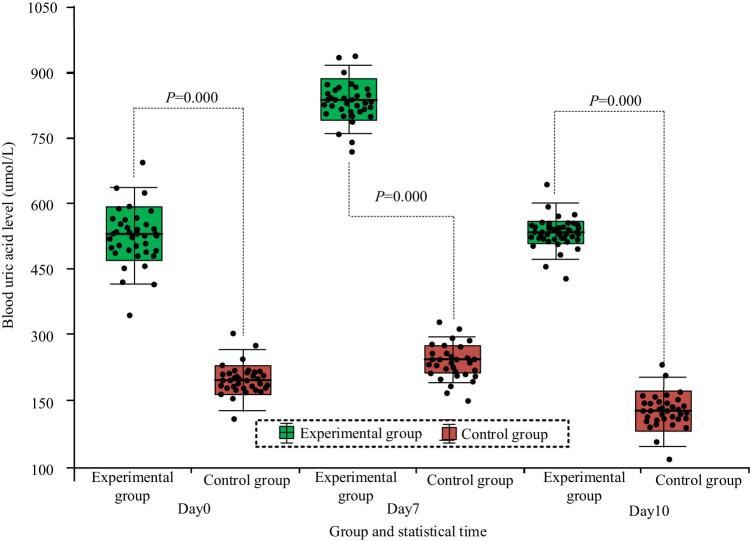
Distribution of blood uric acid data at multiple times in two groups of mice.

The distribution of blood uric acid data at multiple times before and after the experiment in two groups of mice is shown in Figure 7. From Figure 7, the blood uric acid data of mice in the EG and CG all conformed to the normal distribution law. As the experiment progressed, the distribution of blood uric acid data in the EG of mice gradually became concentrated.

### 3.6 Comparison of HOMA-IR index in each group of mice

The HOMA-IR index data of two groups of mice at multiple times before and after the experiment were compared, and the statistical outcomes are shown in Table 11. From Table 11, it can be seen that at each time before and after the experiment, the HOMA-IR index *T*-test *P*-values of the two groups of mice were both less than 0.05, indicating that it had *P* < 0.05. Moreover, at any testing time, the HOMA-IR index of the EG was generally higher than that of the CG.

**Table 11:** Comparison of HOMA-IR index levels between two groups of mice before and after the experiment

Statistical items	EG	CG	Maximum and minimum values	*T*	*P*
Day0	1.48 ± 0.14	1.19 ± 0.13	1.25,0.99	6.788	0.000
Day7	1.51 ± 0.16	1.20 ± 0.12	1.22,1.01	5.492	0.000
Day10	1.78 ± 0.17	1.45 ± 0.14	1.56,1.23	7.541	0.000
Day13	1.96 ± 0.20	1.52 ± 0.15	1.74,1.32	6.946	0.000
Day16	1.84 ± 0.15	1.43 ± 0.12	1.64,1.20	4.339	0.000
Day19	1.75 ± 0.13	1.30 ± 0.14	1.53,1.08	5.267	0.000

### 3.7 Correlation analysis and comparison of various indicators

**Figure 8:**
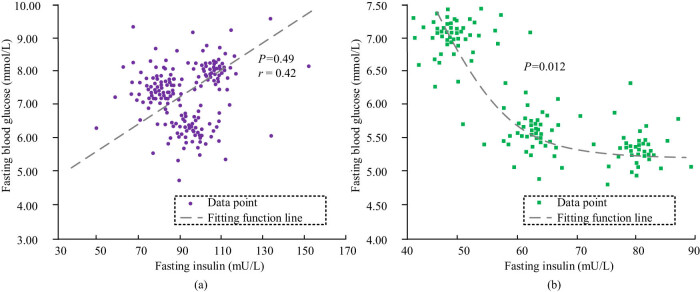
Correlation analysis of fasting blood glucose and fasting insulin in mice of two groups after experiment. (a) EG. (b) CG.

The correlation analysis of the fasting blood glucose and fasting insulin values of the two groups of mice at each time of Day0, Day7, and Day10 after the start of the experiment is shown in Figure 8. From the observation of Figure 8, the fitting curve *P* value between the fasting blood glucose and fasting insulin values of the EG of mice after the injection intervention was 0.49, which was far greater than the significance level of 0.05, and there was no obvious correlation between the data. However, the *P* value of the fitting curve between the fasting blood glucose and fasting insulin values of the CG mice after the injection intervention was 0.012, which was less than the significant level of 0.05, suggesting that there was a significant power function type correlation between the data.

**Figure 9:**
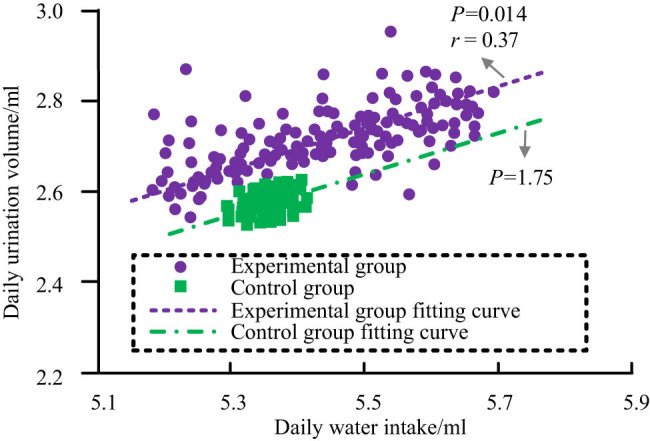
Correlation analysis of water consumption and urination in two groups of mice experiments.

Comparing the correlation between water consumption and urination during the experiment between the two groups of mice, the analysis results are shown in Figure 9. From Figure 9, it can be observed that the *P*-value of the correlation between water consumption and drainage in the CG mice was 1.75, greater than 0.05, indicating that the correlation was not significant. The *P*-value of the correlation between water consumption and drainage in the EG mice was 0.014, less than 0.05, and the correlation coefficient was 0.37, indicating a significant positive correlation between the two.

## 4 Discussion

Research data show that with the advancement of living standards and changes in lifestyle, the incidence rate of HUA has gradually increased at home and abroad, and is affected by age, gender, region, race, lifestyle, and other factors. An analysis denotes that the prevalence of HUA in males and females in China is 21.6 and 8.6%, respectively, which is close to the level of developed countries. HUA will raise the risk of gouty arthritis and chronic kidney disease, and is also closely related to hypertension, hyperlipidemia, obesity, cardio cerebral vascular disease, etc. [15,16]. Therefore, improving people’s understanding of HUA and its related diseases has certain medical and health values. Diabetes is a chronic disease that seriously affects human health. With the aging population, urban and rural development and lifestyle changes, the prevalence of diabetes has gradually increased [17,18]. Compared with 30 years ago, the prevalence of diabetes in China is increasing. Currently, the prevalence of diabetes in China is more than 11%, and the prevalence of pre-diabetes is more than 50%. The number of diabetes patients and pre-diabetes patients in the world reaches 1.139 billion and 439.4 billion, respectively [19,20]. Diabetes and its complications, such as great vessels diseases (coronary heart disease, stroke) and microvascular diseases (diabetes nephropathy, diabetic retinopathy, etc.), pose a serious threat to human health [21,22].

Relevant studies show that the incidence rate of diabetes in patients with HUA is significantly higher than that in people with normal uric acid level [23,24]. Estiverne and Mount also showed that the incidence rate of diabetes in patients with HUA was 2–3 times higher than that in people with normal uric acid level [25,26]. Some clinical studies also found that the proportion of diabetes patients with HUA was significantly higher than that of normal blood glucose people, especially the serum uric acid level of type 2 diabetes patients was higher [27]. All these suggest that HUA may be connected to the occurrence and development of diabetes. A considerable portion of previous studies cannot confirm that blood uric acid levels have a significant impact on insulin sensitivity. Because uric acid has the oxidation effect of promoting adipose precursor cell to differentiate into adipocytes, adiponectin, as a specific insulin sensitizer for adipocytes, uric acid can play a role in reducing the synthesis of adiponectin and reducing insulin sensitivity, thus affecting the glucose metabolism of organisms. In addition, uric acid can also cause chronic inflammatory reaction and increase the production of chemotactic protein-1 factor of monocyte. On the contrary, the pathogenesis of type 2 diabetes is the increase in insulin concentration caused by prophase insulin resistance. As the disease progresses, pancreatic islet function gradually declines. In the state of hyperinsulinemia, insulin resistance will increase the synthesis of fat in the liver, further affect the purine production pathway, and eventually lead to increased uric acid production. High levels of insulin can stimulate the exchange of sodium ions and hydrogen ions in renal tubules, thus increasing the reabsorption of uric acid in renal tubules, making the blood uric acid level exceed the normal range, which may eventually lead to the occurrence of HUA.

At the beginning of this study, the targeted gene modification technology was used to knock out the Urate oxidase gene of C57BL/6J mice, and a spontaneous HUA mouse model was created. In the experiment, the homozygous and wild-type C57BL/6J mice with urate oxidase gene knockout were selected to construct the EG and the CG, and the mice in both groups received multiple intraperitoneal injections of small-doses of STZ solution. The changes in blood sugar and pancreatic β cell function were observed and compared between the EG and the CG mice. The experimental results showed that the level of fasting blood glucose between the two groups before the injection of STZ solution had no significant difference (*P* > 0.05). The plasma insulin levels of the EG mice were higher than those of the CG mice, with significant difference (*P* < 0.05). At the same time, the HOMA-IR index and fasting blood uric acid level of the EG mice were overall higher than those of the CG mice, with significant difference (*P* < 0.05). Starting from the 7th day of injection of STZ solution, the random blood glucose levels of the EG mice began to be higher than those of the CG mice, and this difference persisted until the end of the experiment, with significant difference (*P* < 0.05). At the same time, it was observed that the water and urine intake of the EG were overall higher than those of the wild-type mice, and with significant difference (*P* < 0.05). However, the food intake and body weight of the EG mice did not significantly increase. The blood glucose levels of the EG mice only increased after the increase in blood uric acid levels, while the CG did not experience a sustained increase in blood uric acid levels, so the overall blood glucose did not increase.

In the study, it was observed that the HOMA-IR index of HUA mice was significantly higher than that of the CG, which was consistent with changes in fasting blood glucose and insulin levels. Specifically, the HOMA-IR index of HUA mice was about 23% higher than that of the CG on the 10th day of the experiment (*P* < 0.05), and their fasting blood glucose levels were also significantly increased (*P* < 0.05). In addition, the insulin levels in HUA mice were significantly higher than those in the CG (*P* < 0.05). These results suggested that HUA may promote insulin resistance by affecting insulin sensitivity, and this effect was closely related to the elevation of blood glucose and insulin levels. Therefore, the management and treatment strategies for HUA should focus on its impact on insulin sensitivity.

During the experiment, fasting insulin and blood uric acid levels were detected, and it was found that the EG mice were bigger than the CG mice, with significant difference (*P* < 0.05). There was a certain positive correlation between blood glucose levels and blood uric acid levels. The research outcomes indicated that after STZ solution damages pancreatic tissue to a certain extent, the phenomenon of blood sugar increase in the EG mice occurred earlier than that in the CG mice. At the end of the experiment, a random decrease in blood glucose and uric acid levels was detected, confirming that the findings of this study were consistent with previous studies [28].

In the HUA group, there was a significant positive correlation (*P* < 0.05) between fasting blood glucose and insulin levels. Specifically, on the 10th day of the experiment, fasting blood glucose levels increased to 8.25 ± 0.84 mmol/L, and insulin levels also significantly increased to higher levels. In contrast, this correlation was weaker and not significant in the CG. This discovery suggests that in environments with HUA, insulin resistance may intensify, leading to increased insulin secretion to maintain blood sugar stability, but the effect is limited, which in turn promotes sustained increases in blood sugar. Therefore, HUA not only directly affects the blood sugar level, but also may accelerate the occurrence and development of diabetes by interfering with the normal physiological function of insulin.

Before the injection of STZ solution, the comparison of fasting insulin level and fasting blood glucose level showed that although the insulin content of the EG mice was high, the fasting blood glucose level was not significantly different from the CG, which may be related to the insulin resistance caused by HUA. In this experiment, the comparison of HOMA-IR index of insulin between the two groups of mice showed that the HOMA-IR index of insulin of EG mice were higher than that of the CG mice, further indicating that HUA may be closely related to insulin resistance, which was also consistent with the conclusions of previous studies [29,30]. However, this study has not yet further explored the specific mechanisms that lead to insulin resistance, which is also a deficiency of the experiment. In the experiment, it was observed that on the 7th day after the injection of STZ solution, the fasting blood glucose level of the mice in the two groups was lower than that before the injection, which may be related to the damage of STZ to the pancreatic tissue structure, resulting in the compensatory increase in insulin secretion by β cells in the islets. However, further experiments are needed to verify the lack of detection of fasting insulin levels on that day. In addition, high levels of uric acid may interfere with the insulin signaling pathway, further exacerbating insulin resistance. HUA can affect insulin sensitivity through various mechanisms, such as promoting oxidative stress and inflammatory responses, which can impair pancreatic β cell function, reduce insulin secretion, and decrease peripheral tissue responsiveness to insulin. However, further experiments are needed to verify the lack of detection of fasting insulin levels [31,32].

On the 10th day of the experiment, the HOMA-IR index of HUA mice was about 23% higher than that of the CG. The potential mechanism of HUA induced insulin resistance may involve oxidative stress and chronic inflammatory response [33]. High uric acid levels promote the differentiation of adipocyte precursor cells into adipocytes, reduce the synthesis of specific insulin sensitizer adiponectin, and thus decrease insulin sensitivity. In addition, uric acid can trigger chronic inflammatory reactions and increase the production of monocyte chemoattractant protein-1 factor. The study showed that the HOMA-IR index of hyperuricemic mice was significantly higher than that of the CG, and with the increase in uric acid level, insulin resistance intensified, thereby promoting the development of diabetes.

HUA significantly affects oxidative stress and inflammation levels in mice. Experimental data showed that the plasma lipid peroxide levels in HUA mice were significantly higher than those in the CG (2.20 ± 0.30 µmol/L vs 1.50 ± 0.20 µmol/L), while the levels of antioxidant enzymes SOD, catalase, and glutathione were significantly reduced. High uric acid may damage endothelial function by promoting oxidative stress and inflammatory response, manifested as increased levels of ADMA and decreased levels of NO and eNOS. These changes are closely related to the development of cardiovascular disease. HUA may increase the risk of cardiovascular disease by increasing oxidative stress and inflammation, accelerating the process of atherosclerosis.

High levels of uric acid can impair pancreatic β cell function, as evidenced by reduced β cell counts and proinsulin expression in HUA mice (CG) compared to the CG (EG). Despite compensatory increases in pancreatic islet area and β cell density in the CG, their function remained impaired. This indicated that uric acid may directly affect the health of β cells and may lead to insulin resistance. The significant correlation between HUA and β cell dysfunction emphasizes that uric acid is a key factor in the development of diabetes reducing uric acid level in patients with HUA and insulin resistance may improve glucose metabolism and prevent diabetes by improving insulin sensitivity and reducing inflammation. High uric acid is associated with oxidative stress and chronic inflammation, which affect the development of diabetes.

In addition, this study also explored an obvious positive correlation between the water intake and urine output of the EG mice, which was consistent with industry experience. However, there was no significant correlation between the water intake and urine output of the CG mice. This is mainly because the range of changes in water intake in the CG during the experiment was significantly smaller compared to the EG, making it difficult for the correlation analysis model to find correlation from the data with unclear change information.

At the end of the experiment, immunohistochemical observation of pancreatic tissue showed that the pancreatic tissue structure of the EG mice was swollen compared to the CG mice, and the number of pancreatic β cells was reduced, with significant difference (*P* < 0.05). However, the mechanism of pancreatic β cell damage in the EG mice is still unclear, and further research is needed, which is also the shortcoming of this experiment.

In conclusion, the study successfully constructed a spontaneous HUA mouse model by knocking out the urate oxidase gene of C57BL/6J strain mice through targeted gene modification technology. The experimental data denoted that for spontaneous HUA mice, the continuous rise of blood uric acid level caused by the disease would lead to the rise of blood sugar content, thus promoting the occurrence of diabetes. Second, the content of uric acid in spontaneous HUA mice was maintained at a high level, which would bring or aggravate pancreatic islets β cell damage. The spontaneous HUA mouse model designed in this study is conducive to improving the authenticity and reliability of the experimental results. Although this study still needs in-depth research on the specific mechanism of HUA leading to insulin resistance, it has provided a basis for further exploration of this issue in the future. These findings suggest that HUA is closely related to the development of diabetes and cardiovascular disease. Therefore, attention should be paid to the management of HUA in clinical practice. The treatment strategy should include lifestyle adjustments such as controlling diet, moderate exercise, limiting alcohol consumption, as well as medication treatment such as using uric acid lowering drugs such as allopurinol and febuxostat. For patients with high cardiovascular risk, blood uric acid levels should be more strictly controlled and combination therapy should be considered. At the same time, the blood uric acid level and other relevant indicators should be monitored regularly to prevent or treat diabetes and cardiovascular diseases related to HUA.
